# Development of breakthrough bleeding model of combined‐oral contraceptives utilizing model‐based meta‐analysis

**DOI:** 10.1002/psp4.13261

**Published:** 2024-11-17

**Authors:** Huili Chen, Dain Chun, Karthik Lingineni, Serge Guzy, Rodrigo Cristofoletti, Joachim Hoechel, Tianze Jiao, Brian Cicali, Valvanera Vozmediano, Stephan Schmidt

**Affiliations:** ^1^ Department of Pharmaceutics, College of Pharmacy, Center for Pharmacometrics and Systems Pharmacology University of Florida Orlando Florida USA; ^2^ Novartis Pharmaceutical Corporation Cambridge Massachusetts USA; ^3^ Pop‐Pharm Pharmacometrics Service Albany California USA; ^4^ Bayer AG Berlin Germany; ^5^ Department of Pharmaceutical Outcomes and Policy, College of Pharmacy University of Florida Gainesville Florida USA; ^6^ Model Informed Development CTI Laboratories Covington Kentucky USA

## Abstract

Breakthrough bleeding (BTB) is a common side effect of hormonal contraception and is thought to impact adherence to combined oral contraceptives (COCs) but respective dose–response relationships are not yet fully understood. Therefore, the objective of this model‐based meta‐analysis (MBMA) was to establish dose–response for COCs containing different progestin/EE combinations using BTB as the pharmacodynamic endpoint. Data from 25 studies containing BTB information of 4 progestins (desogestrel, drospirenone, gestodene, and levonorgestrel) in combination with ethinyl estradiol (EE) at various dose levels was used for this analysis. The results of our MBMA show that BTB is significantly increased upon initiation of COC use but subsides over time. The time needed for BTB to return to baseline depends on the EE dose and differs marginally between progestins during the initial months of use at the same EE dose. BTB typically returns to baseline within 3 months at the highest (30 μg) dose, whereas it can take significantly longer to reestablish a regular bleeding pattern at lower EE doses (15 and 20 μg), irrespective of the progestin used. The dose–response relationships established for BTB across different progestin/EE combinations can now be used to support the selection of optimal COC dosing/treatment regimens and serve as the scientific basis for evaluating the impact of clinically relevant factors, including drug–drug interactions and demographics, on BTB.

**Study Highlights**

**WHAT IS THE CURRENT KNOWLEDGE ON THE TOPIC?**

BTB is thought to contribute significantly to COC non‐adherence and discontinuation and, thus, result in an increased risk of unintended pregnancies. While EE is known to mitigate BTB, respective dose–response relationships are not yet fully understood.

**WHAT QUESTION DID THIS STUDY ADDRESS?**

What is the dose–response relationship between progestins/ethinylestradiol and breakthrough bleeding?

**WHAT DOES THIS STUDY ADD TO OUR KNOWLEDGE?**

We characterized the dose–response relationship of fourdifferent progestins at various EE doses and BTB. The time needed for BTB to return to baseline following COC use is significantly impacted by the EE dose, with higher EE doses (30 μg) leading to faster resolution (within 3 months). There is a significant correlation between progestin and EE dose, which impacts the decline in BTB during the initial months of COC use.

**HOW MIGHT THIS CHANGE DRUG DISCOVERY, DEVELOPMENT, AND/OR THERAPEUTICS?**

The findings of this study can be used to select optimal COC dosing/treatment regimens and serve as the scientific basis for evaluating the impact of clinically relevant factors, including drug–drug interactions and demographics, on BTB.

## INTRODUCTION

Combined oral contraceptives (COCs) play an essential role in preventing unintended pregnancies among women of childbearing age. However, the efficacy of COCs can be significantly reduced by non‐adherence and discontinuation, which are thought to contribute to 20% of the 3.5 million annual unintended pregnancies in the United States.^1^ Approximately 61% of these unintended pregnancies occur in women who discontinue COCs due to side effects and do not immediately switch to another reliable contraceptive method.^2^ Literature evidence suggests that breakthrough bleeding (BTB), defined as unscheduled vaginal bleeding occurring outside the regular bleeding period associated with hormonal contraceptive administration that requires the use of more than one tampon or sanitary pad per day, is one of the most common side effects impacting adherence to COCs.^3^ Sufficient consideration of BTB is consequently important when developing new hormonal contraceptives or optimizing the use of existing COCs.^4^

COCs usually follow a 21/7 regimen with 21 days of active pills and a 7‐day placebo or pill‐free interval for withdrawal bleeding, mimicking normal menstruation, although new regimens like 24/4 and 26/2 have recently been developed. They typically consist of two components, a progestin and an estrogen. Most progestins achieve their contraceptive efficacy through the inhibition of gonadotropin‐releasing hormone (GnRH) secretion by the hypothalamus and the subsequent suppression of luteinizing hormone (LH) and follicle‐stimulating hormone (FSH) release by the pituitary gland. This hormonal modulation disrupts the menstrual cycle, effectively preventing ovulation. Concurrently, progestins impair endometrial vascular integrity, which can result in breakthrough bleeding (BTB).^5^ Estrogens, typically ethinyl estradiol (EE), are used in COCs to control BTB by maintaining endometrial integrity.^6^, ^7^ However, EE is thought to also contribute to the contraceptive activity by inhibiting FSH release. It should be noted that the occurrence of BTB is time‐variant. BTB is typically highest within the first month of COC initiation but is thought to subside within 3 months.^5^

**FIGURE 1:**
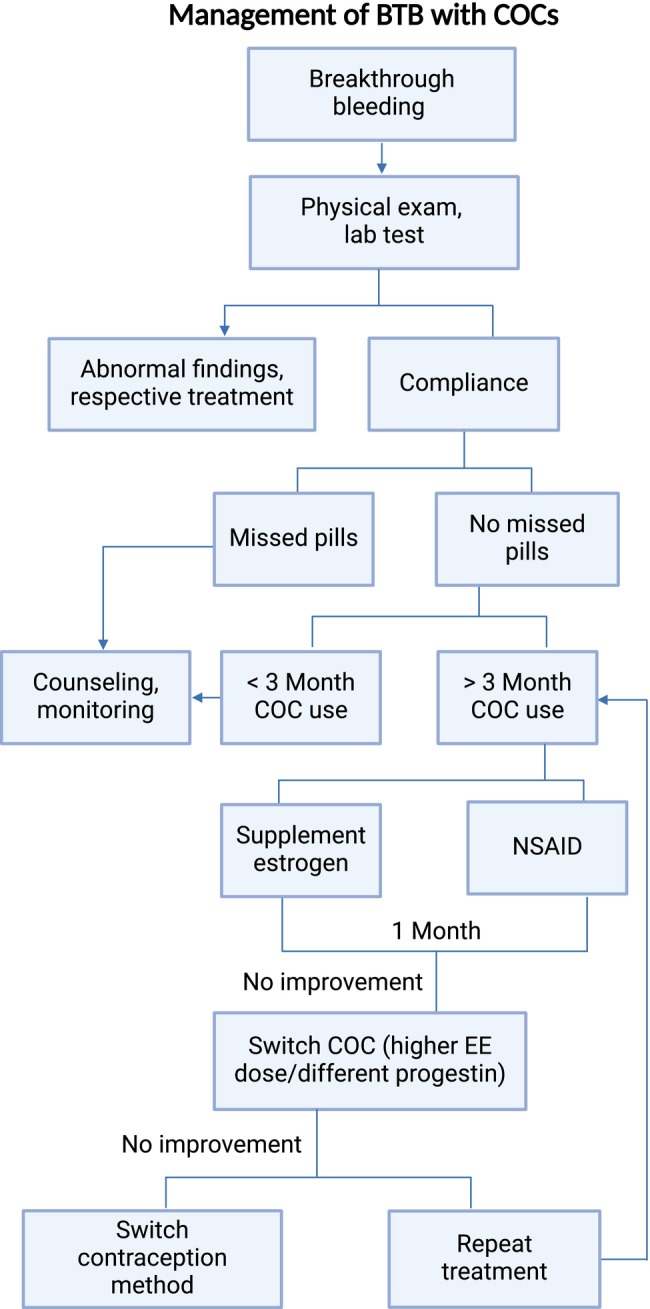
Evaluation and management algorithm for BTB in COC users. NSAID, non‐steroidal anti‐inflammatory drugs.

The management of BTB in women using COCs can be challenging. According to the current clinical management plan shown in Figure 1, physical examinations and laboratory tests are recommended for women experiencing BTB while using COCs to rule out any underlying abnormalities, followed by an assessment of adherence to treatment.^5^ If BTB persists beyond 3 months in COC treatment‐adherent women, the use of supplemental estrogen or non‐steroidal anti‐inflammatory drugs (NSAIDs) may be required to alleviate symptoms. Should BTB still persist, a switch to a different progestin or an increase in the EE dose is recommended. However, it is currently unclear which progestin should be switched to and what the optimal EE dose is. Therefore, we set out to develop a quantitative framework for BTB, which integrates information on progestin type, progestin dose, EE dose, as well as subject demographics to inform the optimal use of COCs and improve clinical outcomes.

The establishment of such a framework is hindered by the inconsistent definitions of BTB across clinical trials and the availability of different formulations and COC dosing regimens, making a head‐to‐head comparison of respective clinical trial data difficult. To overcome this challenge, a model‐based meta‐analysis (MBMA) was employed, which allows for integrating data from different studies, thus enabling simultaneous comparison of varying progestin/EE combination‐ and dosing regimens. The statistical rigor of MBMA also allows for identifying covariate relationships, which can be used to optimize treatment and dosing regimens. Our group has previously established dose‐exposure‐response relationships of levonorgestrel and drospirenone via MBMA and physiological‐based pharmacokinetics (PBPK) modeling with Pearl Index (PI) as an efficacy endpoint allowing to define systemic exposure thresholds needed to achieve PI values used in drug development and regulatory evaluation.^8^, ^9^ The work presented in this manuscript complements these prior analyses by establishing respective dose–response relationships using BTB as endpoint. Once developed and verified, derived dose‐exposure‐response relationships for efficacy endpoint (PI) and side effect (BTB) can be used to guide the selection of optimal COC dosing and treatment regimens.

## METHODS

**FIGURE 2:**
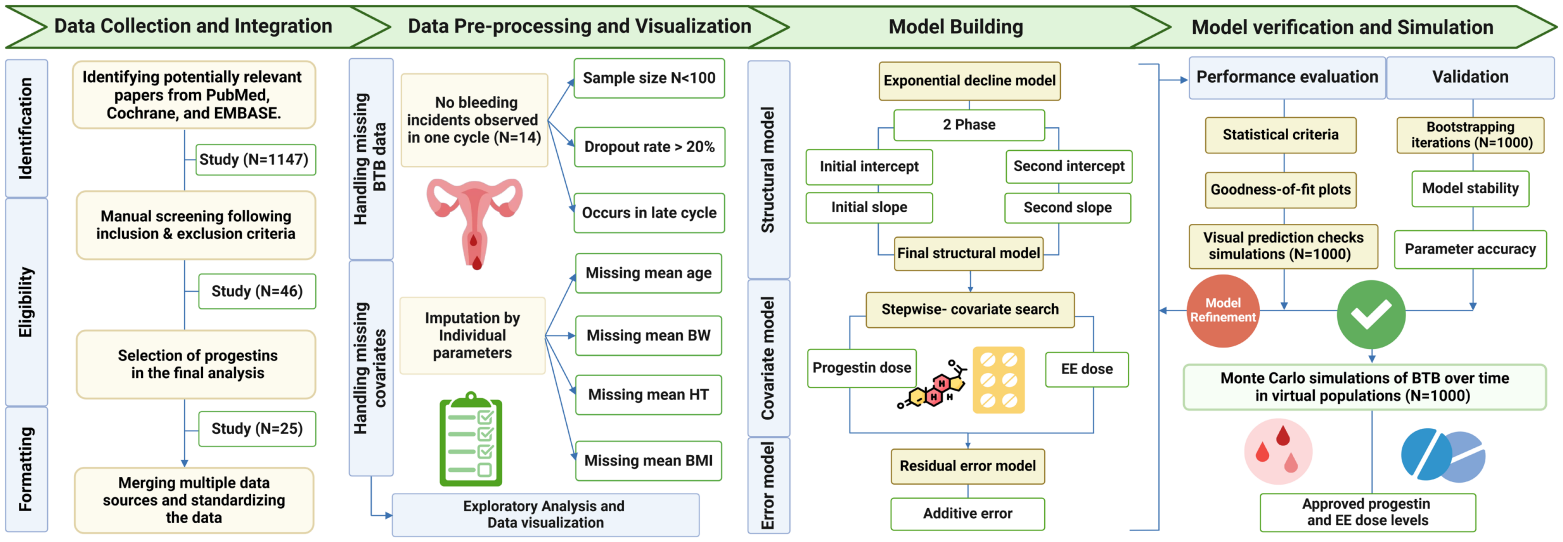
Analysis workflow.

We developed a comprehensive MBMA to evaluate breakthrough bleeding associated with combined oral contraceptives according to the workflow outlined in Figure 2. First, we conducted a comprehensive literature review to extract relevant BTB data, followed by manual screening using predefined inclusion and exclusion criteria. Progestins of interest were selected based on data availability, ensuring sufficient observations for analysis. To address missing data, we applied imputation techniques for both BTB observations and covariates. Second, we developed a structural model and assessed the impact of available covariates on changes in BTB over time. Once developed and verified, the model was then used in simulations to compare the impact of different progestin/EE combinations on BTB.

### Data

A systematic literature search was performed in conjunction with Excelra Knowledge Solutions Pvt. Ltd. (Hyderabad, India) across multiple databases, including PubMed, Cochrane, and EMBASE, to identify COC studies for which BTB was reported. Details of this literature search have been previously reported in Lingineni et al.^8^ Briefly, relevant information, including definitions of BTB, subject demographics, trial methodologies, progestin types (levonorgestrel (LNG), drospirenone (DRSP), gestodene (GSD), desogestrel (DSG), norethindrone, dienogest and norgestrel), progestin dose, EE dose, and corresponding BTB outcomes, were extracted and screened for consistency. The inclusion criteria encompassed any bleeding episodes during active hormone intake, except those that occurred within the initial 7 days of the first cycle or from days one to four of subsequent cycles. Exclusion criteria included alternative COC indications, non‐observational studies, absence of active treatment with specified hormones, multiphasic dosing, deviations from the typical 21/7 dosing regimen, and review articles. A summary of the inclusion and exclusion criteria, along with key search terms, such as hormonal contraceptives and bleeding outcomes (e.g., breakthrough bleeding, intermenstrual bleeding), are available in Table S1.

For cycles with fewer than 100 subjects, where the probability of observing BTB events was <1%, observations were marked as missing. To address this data challenge, the natural occurrence of unscheduled bleeding, which occurs before the initiation of hormonal contraceptive treatment, was used as a baseline instead. Data from seven internal Bayer studies showed that unscheduled bleeding can occur even in the absence of contraceptive drugs, with an observed baseline rate of 1.68%. This baseline, calculated from pre‐treatment data involving 44,420 subjects, was used to impute missing BTB data by assigning half of the baseline value, ensuring accurate slope estimation.^9^, ^10^

The impact of missing covariates was systematically assessed by comparing three approaches for handling missing covariate data: (1) single imputation by median, (2) multiple imputation by chained equations, and (3) imputation by individual parameter (IIP) to estimate the missing covariate values. The latter assumes a log‐linear relationship between the individual covariate and parameter, which can be expressed as:

(1) $$\ln\left(\mathrm{CoV}\right)=k_{0}+k_{1}\cdot \theta_{i}+\varepsilon ,\;\varepsilon \sim N\left(0,\sigma^{2}\right)$$

where *k₀* and *k₁* is the intercept and slope coefficient associated with the log‐linear regression function; $\theta_{i}$ is the individual parameter of ith study obtained from the base model, and *ε* is the residual error. After comparing the performance of all three methods, IIP was chosen as the final approach for its ability to handle missing covariates without relying on other demographic variables, while maintaining the correlation between parameters and covariates. An additional sensitivity analysis further confirmed that this approach provided the most robust and consistent results.

After establishing the structural model, the covariates from individual studies were paired with the estimated structural parameters, and a log‐linear regression was fitted. *k*₀ and *k*₁ were calculated using the least squares method, minimizing the sum of squared residuals between the observed and the predicted $\ln\left(\mathrm{CoV}\right)$ values from the regression model. To handle missing covariates, the most significant relationship between the available covariates and the estimated structural parameters was identified. This relationship was then used to estimate the missing covariates based on the values of the estimated individual parameter.

### Model‐based meta‐analysis

An MBMA was performed, where different structural models with mono‐phasic, bi‐phasic, and tri‐phasic, exponential decline in BTB over time were evaluated. The method of residuals was applied to obtain initial estimates for employed macro‐constants.

To accurately estimate the initial BTB intercept parameter, the time of the first observation was set to 0, corresponding to the completion of one full cycle of COC treatment. This adjustment was made to account for the substantial variability in BTB observed following the initial cycle. By setting the time to 0 at the first observation, we ensured a more reliable and consistent estimation of the initial BTB induction across all studies, reducing potential bias introduced by early‐phase fluctuations.

Population parameters were assumed to be log‐normally distributed and characterized using Equation 2,

(2) $$\theta_{i}=\theta_{\mathrm{pop}}\cdot e^{\eta_{i}}$$

where $\theta_{i}$ represents the study‐specific parameter of the ith study, $\theta_{\mathrm{pop}}$ represents the typical (population) value of the parameter, and $\eta_{i}$ denotes the study deviation from this average value. Covariances among the inter‐study variance (ISV) were also estimated to account for potential correlations between parameters. Different error models, including additive, proportional, and combined‐additive and proportional residual error models, were explored to characterize residual unexplained variance (RUV) was explored by testing.

Clinically significant and mechanistically plausible covariates, including intrinsic factors, such as study‐level average demographics (age, weight, height, body mass index [BMI]), and extrinsic factors, such as progestin type, progestin dose, and EE dose were included in the covariate analysis. Given that progestins differ in molecular weight, molar doses were used in the analysis to mitigate collinearity issues. Pearson's correlation coefficients were calculated for covariate pairs. Covariates with an absolute correlation >0.6 were excluded from further analysis. ANOVA (analysis of variance) was used to test significant differences (*p*‐value < 0.05) of the continuous variable across the different categorical variable. A stepwise covariate modeling approach assessed the impact of various covariates on model parameters with the forward and backward thresholds for the likelihood ratio test (LRT) that were set at 0.01 and 0.001, respectively.

The final BTB model was developed by evaluating the precision of parameters, objective function value (OFV), and graphical examinations of goodness‐of‐fit plots. The precision of the parameter estimates for the final model was assessed via bootstraps using 1000 resampling iterations. Diagnostic plots, including observations versus individual predictions (IPRED), observations versus population predictions (PRED), conditional weighted residuals (CWRES) versus PRED, CWRES versus TIME, individual weighted residuals (IWRES) versus IPRED, and IWRES versus time, were generated for model comparison. Furthermore, individual fitting and visual predictive check (VPC) plots evaluated the model's performance and ability to capture observed data accurately, using the final model estimates, 1000 Monte Carlo simulation replicates were generated for BTB over time profiles of COCs and compared with observed data in a VPC. The simulated BTB change over time along with 10th, 50th, and 90th percentiles, and their respective 95% confidence intervals were computed and overlaid for comparison with observed data points.

The final model was used to simulate BTB for a subset of FDA‐approved combinations of progestins and EE at dosage levels, including DSG/EE (150/20 or 150/30 μg), DRSP/EE (3000/20 or 3000/30 μg), GSD/EE (60/15, 75/20 or 75/30 μg), and LNG/EE (100/20 or 150/30 μg).^11^ For each combination of progestin and EE, 1000 Monte Carlo simulation sets were generated. Summary statistics of BTB after 1, 3 and 4 months of COC treatment at approved progestin/EE dose levels including minimum, maximum, mean, standard deviation, median, and 25th, 50th, and 75th percentiles of simulated BTB values were generated. The time required to return to baseline unscheduled bleeding levels was also computed for comparison across the approved COCs using individual parameters from simulations.

The model was developed in NONMEM (version 7.5.0; Icon Development Solutions, Ellicott City, MD) with first‐order conditional estimation with interaction (FOCEI) algorithm using Finch Studio interface (version 1.5.0; Enhanced Pharmacodynamics, LLC, Buffalo, NY) along with Perl‐speaks‐NONMEM (version 5.4.0;) to achieve stable final parameter estimates. Simulations were performed using Simulx (version 2024R1; Simulations Plus, Inc., Lancaster, CA). Statistical tests and simulation plots were generated using R (version 7.4.4) and Python (version 3.12).

## RESULTS

### Data

The systematic literature search identified 1147 initial papers using the search criteria outlined in Table S1. After applying the inclusion and exclusion criteria, a total of 46 studies containing BTB data were considered for further analysis. Progestin‐only pill (POP) data was not used for model development due to a limited number of studies and observations. However, these POP data suggested a high prevalence of BTB following treatment initiation, which only slowly subsided over time.^12^, ^13^ Longitudinal data for 4 COCs (DSG/EE, DRSP/EE, GSD/EE, and LNG/EE) was deemed sufficient to achieve the objective of this MBMA. There was only limited BTB data for other progestins. These progestins were consequently excluded from this MBMA to improve the robustness of the analysis. The final MBMA dataset contained BTB data from 25 studies, 33 unique treatment arms, and 228 observations of these 4 progestins at various progestin/EE dose combinations which are summarized in Table 1 and Table S2.

**TABLE 1:** Overview of the combined‐oral contraceptive trials included in this model‐based meta‐analysis.

Progestin type	Progestin dose (μg)	EE dose (μg)	No. of trials	Median No. of subjects	Median No. of observations	Median treatment duration (month)	Mean age (Min, Max) (year)	Mean BW (Min, Max) (kg)	Mean HT (Min, Max) (m)	Mean BMI (Min, Max) (kg/m^2^)
DSG	150	20	3	338	12	10.27	31.17 (25.1, 40.2)	59.30 (59.3, 59.3)	N/A	22.10 (22.1, 22.1)
		30	4	327	4	6.84	25.97 (25.3, 26.4)	58.30 (56.7, 59.9)	166.20 (166.20, 166.20)	N/A
DRSP	3000	20	1	461	26	23.34	24.60 (24.6, 24.6)	63.40 (63.4, 63.4)	167.98 (167.98, 167.98)	22.40 (22.4, 22.4)
		30	5	203	3	5.00	25.62 (22.5, 27.2)	61.20 (52.8, 68.3)	162.67 (154.80, 167.10)	25.20 (22.8, 27.6)
GSD	60	15	3	94	6	4.67	27.58 (25.0, 30.1)	53.70 (53.7, 53.7)	157.30 (157.30, 157.30)	21.40 (21.4, 21.4)
	75	20	1	740	12	10.27	25.50 (25.5, 25.5)	59.20 (59.2, 59.2)	N/A	N/A
		30	4	309	6	4.67	26.27 (25.6, 27.2)	59.37 (57.1, 61.2)	N/A	N/A
		35	1	5602	6	4.67	16.40 (16.4, 16.4)	57.00 (57.0, 57.0)	N/A	N/A
LNG	100	20	5	380	6	4.67	26.52 (25.3, 28.5)	63.20 (62.7, 64.2)	167.30 (167.00, 167.80)	22.80 (22.8, 22.8)
	150	30	6	176	4	6.07	26.19 (25.5, 26.9)	55.18 (46.7, 64.4)	154.67 (154.60, 154.74)	22.08 (22.1, 22.1)
Total	6	4	33	308	6	4.67	26.38 (16.4, 40.2)	59.17 (46.7, 68.3)	162.79 (154.60, 167.98)	23.03 (21.4, 27.6)

The demographic data before and after imputation (Table S2) exhibited similar minimum, mean, median, and maximum values. IIP showed unbiased imputed missing values within the observed value range compared to single and multiple imputations. It also demonstrated the advantage of not relying on additional covariates, particularly when those covariates are unavailable in some studies, making it a more robust method for handling missing data in such scenarios.

### Model‐based meta‐analysis

Exploratory plots showed a substantial increase in BTB from baseline upon COC initiation (up to ~4.5‐fold), followed by a two‐phasic decline with a rapid decline during the first 2–3 months and a much slower decline thereafter. POPs showed a significantly higher increase in BTB from baseline (up to ~29.2‐fold).^12^ The addition of EE resulted in decreased BTB following treatment initiation and subsequent cycles compared to POP drug products. The findings of this exploratory analysis translated into the final 2‐compartment model (Equation 3),

(3) $$\mathrm{BTB}=Ae^{-\mathrm{\alpha x}}+Be^{-\mathrm{\beta x}}$$

where *A* is the intercept of the initial phase, α is the slope of the rapid, initial decline of BTB, *B* is the intercept of the second phase, and β is the slope of the second, slower phase of decline. In comparison, the use of a 1‐compartment model resulted in biased estimates at later time points, whereas the use of a 3‐compartment model did not significantly improve the model fit. The use of a 3‐compartment model also resulted in model instability and shrinkage.

The results of the covariate analysis show that the EE dose has a significant impact on B. Mean BMI showed a significant effect on A. However, BMI was not included in the final covariate model due to the limited BMI range, with the majority of values laying in a relatively narrow range (21.4–23.5 kg/m^2^), with the exception of DRSP/EE 3000/30 μg group, where subjects had a significantly higher BMI of 27.6 kg/m^2^ (Table 2).

**TABLE 2:** Parameter estimates for the MBMA of the final BTB model.

Parameter	Estimate	RSE (%)	Bootstrap (*N* = 1000)		
			Median	Lower 95% CI	Upper 95% CI
Fixed effect parameters					
A	0.0383	21.7	0.037	0.026	0.050
α	0.922	35.4	1.050	0.032	1.813
B	0.0134	34.2	0.014	0.006	0.021
β	0.0524	32.5	0.056	0.028	0.077
EE dose on B	−2.45	32.6	−2.422	−3.873	−1.028
Progestin dose by MW on α	0.576	22.4	0.573	−0.231	1.383
Random effect parameters					
ISV‐ A	0.824 (113% CV)	40.3	0.859	0.255	1.392
ISV‐ α	0.959 (127% CV)	42.3	0.987	−0.095	2.012
ISV‐ B	0.223 (49.9% CV)	67.6	0.205	−0.124	0.569
ISV‐ β	0.138 (38.5% CV)	84.3	0.126	−0.009	0.284
Residual unexplained variance					
Unweighted additive error	0.0410 (20.2% CV)	35.2	0.035	0.024	0.058

Abbreviations: CI, confidence interval; ISV, inter‐study variance; RSE, relative standard error; RUV, residual unexplained variance.

The molar progestin dose had initially a significant impact on α but lost statistical significance once EE dose was added as a significant covariate on B. Despite that, we decided to retain the molar progestin dose as a covariate on α due the pharmacological interplay between progestin and EE. Respective covariate relationships were included in the model as follows:

(4) $$\log\;\left(A_{i}\right)=\log\left(\theta_{A,\mathrm{pop}}\right)+\eta_{A,i}$$

(5) $$\begin{array}{c} \log\;\left(\alpha_{\text{Progetsin}\;\mathrm{AMT},i}\right)=\log\left(\theta_{\alpha ,\mathrm{pop}}\right)\\ +\log\;\left(\frac{\text{Progestin}\;\mathrm{AMT}\;}{0.48}\right)\cdot \theta_{\text{Progestin}\;\mathrm{AMT},\alpha }+\eta_{\alpha ,i} \end{array}$$

(6) $$\begin{array}{c} \log\;\left(B_{\mathrm{EE}\;\text{dose},i}\right)=\log\left(\theta_{B,\mathrm{pop}}\right)+\log\;\left(\frac{\mathrm{EE}\;\text{dose}}{30}\right)\cdot \theta_{\mathrm{EE}\;\text{dose},B}\\ \;+\eta_{B,i} \end{array}$$

(7) $$\log\;\left(\beta_{i}\right)=\log\;\left(\theta_{\beta ,\mathrm{pop}}\right)+\eta_{\beta ,i}$$

where θ represents the coefficients associated with the fixed effects; i is an index denoting the individual parameter; pop is an index denoting the typical value across the entire population and η denotes the random effect that captures ISVs. An additive error model was deemed appropriate due to the lowest OFV and improved structural and variance parameter estimates.

The fraction of subjects experiencing BTB is described in Equation 8,

(8) $$Y_{ijk}=F_{ijk}+\epsilon_{ijk}$$

where $Y_{\mathrm{ijk}}$ represents the observed BTB in the *k*th treatment arm of ith study at the *j*th time. $F_{\mathrm{ijk}}$ represents the corresponding model‐predicted BTB and $\varepsilon_{\mathrm{ijk}}$ represents additive residual error. The distribution of the residual error can be characterized as follows

$$\varepsilon_{\mathrm{ijk}}\sim N\left(0,\frac{\sigma^{2}}{N_{\mathrm{ik}}}\right)$$

where $\;N_{\mathrm{ik}}$ is the number of individuals in treatment arm *k* of study *i*.

**FIGURE 3:**
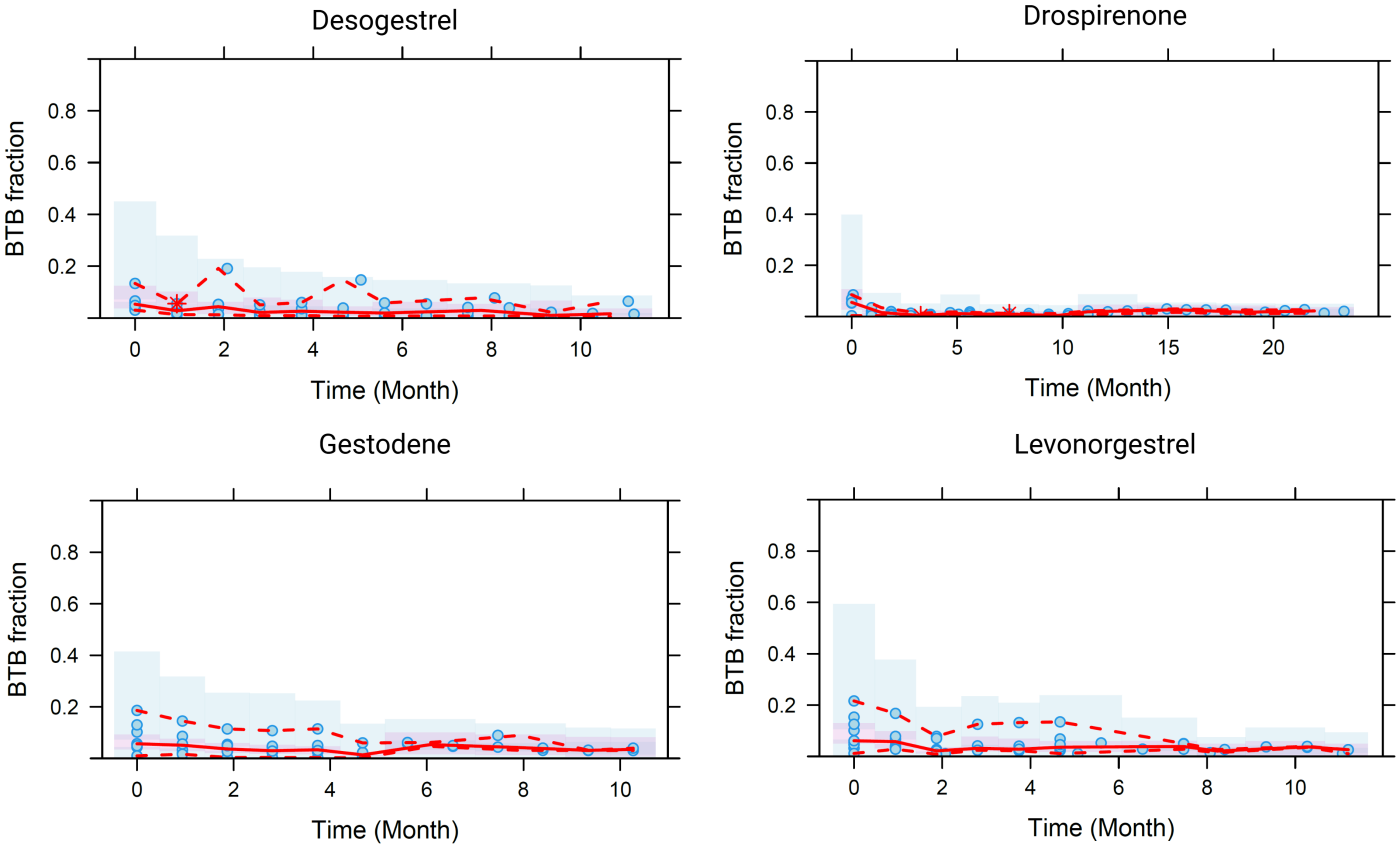
Visual predictive check of the final BTB model: Solid dots represent the observed data, and the color represents the progestin type and EE dose level. The *y*‐axis represents the fraction of women who experienced BTB whereas the *x* axis is expressed as time since the first observation, starting 1 cycle after the initiation of treatment. The lines denote the 10th (dashed), 50th (solid), and 90th (dashed) percentiles of the observed data. Shaded areas indicate the 95% confidence interval of 10th (light blue), 50th (light purple), and 90th (light blue) model predicted percentiles.

The robustness of the model, its structural parameters, ISV, standard errors, and parameter correlations were systematically evaluated. Median parameter values obtained from bootstrap analysis were in good agreement with the respective population estimates of the final model, with the narrow 95% CIs demonstrating good precision, as shown in Table 2. The relative standard error (RSE) values for the ISVs of parameter *A* and α suggested high uncertainty in the extent of initial BTB and its initial, rapid decline across studies. Diagnostic plots (Figure S1) and VPC (Figure 3) showed no significant bias or model misspecification. Observations of all progestin/EE combinations were contained in the respective prediction intervals, except those for DRSP/EE 3000/30 μg.

**FIGURE 4:**
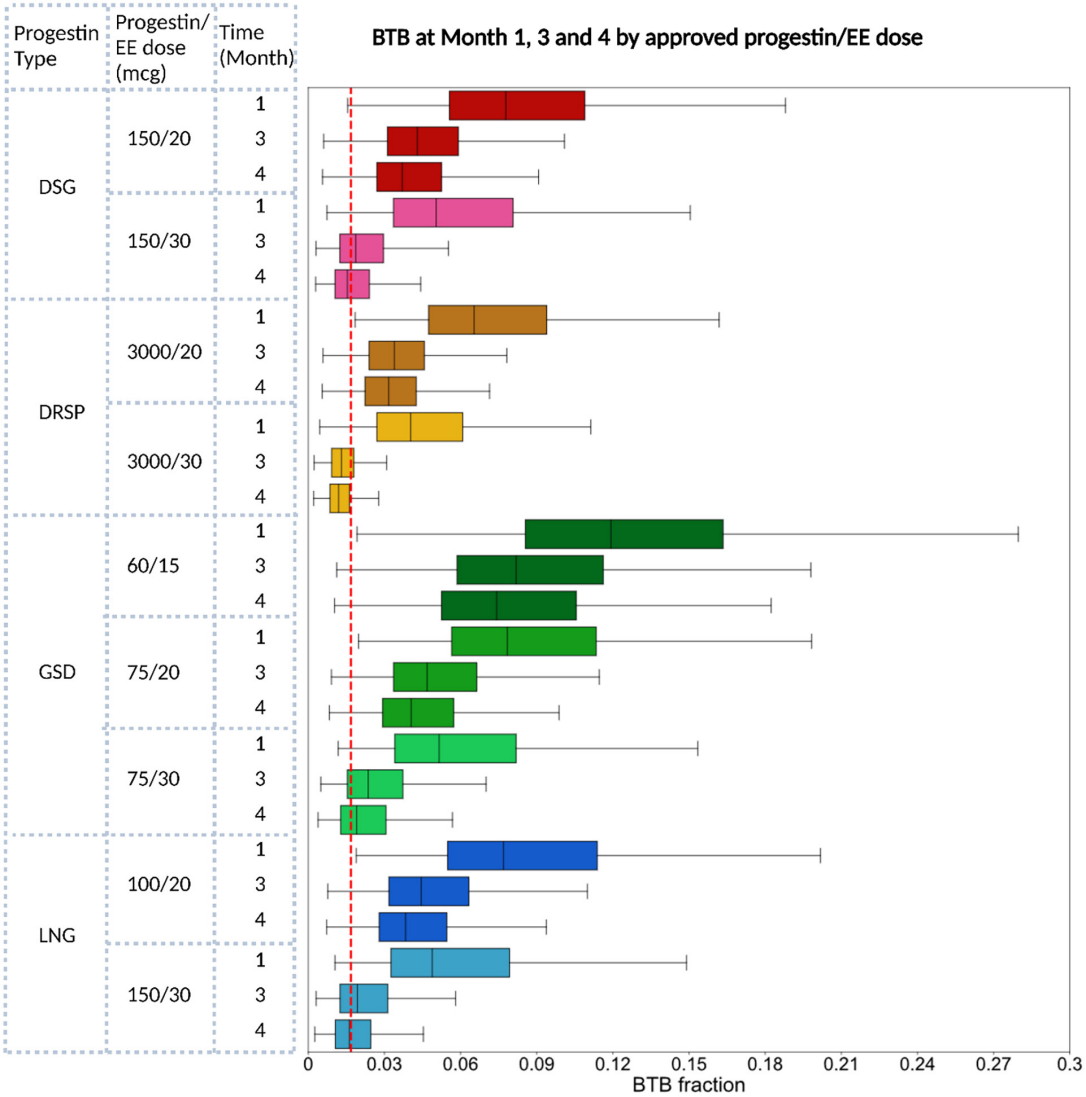
Change in BTB over time upon COC treatment initiation, stratified by treatment regimen and time after initiation of COC (months 1, 3, and 4). The red dashed line represents the baseline BTB.

BTB simulation results indicate that the EE dose significantly impacted BTB over time as shown in Figure 4 and Table S3 for the different progestin/EE combinations. Following the initial treatment month with COCs, median BTB was highest for GSD/EE 60/15 mcg (11.9%) but significantly (*p* < 0.05) lower for other COC drug products, e.g. GSD (7.8%), DSG (7.8%), LNG (7.7%), and DRSP (6.5%), in the presence of the comparatively higher 20 mcg EE dose. The incidence of BTB further decreased once the EE dose was further increased to 30 mcg: GSD (5.2%), DSG (5.0%), LNG (4.9%), and DRSP (4.0%). After 4 months, there were < 1% differences in BTB between the four different progestin products that contained the same EE dose. The time needed for BTB to return to baseline was impacted by both the progestin type and the EE dose, as detailed in Table S4. For COCs containing 30 μg EE, the median time to completely return to baseline was the longest for GSD, with a median duration of 4.8 months, compared to other progestins DSG (3.4 months), LNG (3.7 months), and DRSP (1.6 months). For COCs containing 20 mcg EE, the median recovery time was GSD (15.9 months), DSG (15.6 months), and LNG (16.0 months), while DRSP had a median recovery time of 14.9 months. Median recovery time was the longest (28.6 months) for the GSD/EE 60/15 mcg formulation.

## DISCUSSION

Our study represents the first successful application of an MBMA to establish a dose–response relationship between COCs containing different progestins/EE doses and BTB. The results of our analysis confirm that progestins induce BTB, which is highest upon COC treatment initiation. The addition of EE provides a dose‐dependent, protective effect against BTB, with higher EE doses resulting in lower BTB rates (e.g., GSD with EE at 15 mcg: 11.9%, 20 mcg: 7.8%, and 30 mcg: 5.2%) after the first month of treatment. The time it takes for BTB to return to baseline depends on the EE dose and varies between progestins at the same EE dose. BTB associated with COC drug products containing 30 μg EE typically returns to baseline within 3‐4 months, except for GSD (4.8 months), whereas it can take much longer for COC drug products that contain 20 μg EE. These findings are in line with the current treatment recommendations for BTB,^5^ which acknowledge that BTB is relatively common within the first 3 months upon treatment initiation, particularly for COC drug products containing lower EE doses (i.e., 15 and 20 μg), and may require counseling if BTB does not subside within 3 months. The findings of our analysis supported the recommendation of supplementing with additional estrogen or switching COCs to a formulation with higher EE content to alleviate BTB because the time it takes for BTB to return to baseline is significantly shorter at higher EE doses, irrespective of the progestin used. In contrast, our analysis does not support switching to a different COC containing the same EE dose, especially after 4 months of COC treatment because our analysis no longer showed significant differences in BTB between progestins at and beyond this time.

We would like to acknowledge that the results of our MBMA hinge on several assumptions and are subject to data limitations. The high RSEs for the ISV on A and α indicate uncertainty in the parameter estimates, which is likely due to the availability of only sparse BTB data right after COC treatment initiation. In addition, we retained progestin dose as a covariate on α despite the lack of statistical significance in the final model because of the correlation between progestin dose and EE dose, that is, the higher the progestin dose, the higher the EE dose. To truly separate the two, dose‐ranging data on progestin‐only and EE‐only regimens would be needed, which were not available for this analysis. We are further aware of the fact that demographic factors, particularly BMI, may influence the extent of BTB but were not able to verify these effects due to the narrow BMI range in the available data. Future studies should consequently aim to include subjects across a broader BMI range to conclusively evaluate its impact on BTB and facilitate recommendations for special patient populations.

Our analysis was further hindered by inconsistent or missing definitions and measurement standards for BTB. Differences in the definition of BTB, such as the criteria for sanitary product use and the inclusion of bleeding from previous cycles, resulted in significant inconsistencies in reported BTB measures. To minimize the impact of these inconsistencies on our analysis, we excluded bleeding events during hormone‐free intervals. In addition, we worked closely with our real‐world evidence colleagues to harmonize the BTB definitions used in our analysis with the respective definitions used in ICD‐9/10 codes. In light of these inconsistent definitions, we support the recommendations from the Society of Family Planning, which advocates for independent assessment of bleeding pattern, flow, and duration to gain a more comprehensive understanding of BTB.^14^

We would also like to note that the current FDA guidance of clinical drug interaction studies with combined oral contraceptives primarily focuses on the risk of reduced contraceptive efficacy due to CYP3A4 induction and associated decreases in progestin concentrations.^14^ It is crucial to better understand the impact of CYP3A4 inhibition and corresponding elevations in progestin and/or EE concentrations on BTB though. An increase in EE exposure has also been associated with an increased risk of venous thromboembolisms (VTEs), particularly at doses exceeding 50 μg EE, while the evidence for sub‐50 μg formulations and different progestin types remains inconclusive.^15^ To better understand this dynamic interplay between different progestins, progestin and EE doses, demographic factors, drug–drug‐interactions, and routes of administration, the use of physiologically‐based pharmacokinetic (PBPK) models may offer an opportunity for translating dose–response relationships derived in this MBMA into respective dose‐exposure‐response relationships that allow for a more direct comparison of these different factors within and across formulations. This approach was used in a previous efficacy analysis to establish a link between dose, systemic exposure, and unintended pregnancies for LNG and DRSP,^9^, ^16^ which can be combined with the current analysis and its proposed PBPK expansion to better define an optimal therapeutic window for COCs, mitigate risk factors, and further improve the clinical use of COCs.

## AUTHOR CONTRIBUTIONS

H.C., D.C., K.L., B.C., S.G., R.C., J.H., T.J., V.V., and S.S. wrote the manuscript. H.C., D.C., K.L., B.C., S.G., V.V., and S.S. designed the research. H.C., D.C., K.L., B.C., S.G., V.V., and S.S. performed the research. H.C., D.C., K.L., B.C., S.G., V.V., and S.S. analyzed the data.

## FUNDING INFORMATION

This project was funded by the Bill & Melinda Gates Foundation, including the current grant INV‐010213 and the previous grant OPP1185454.

## CONFLICT OF INTEREST STATEMENT

J.H. is an employee of Bayer AG. All other authors declare no competing interest in this work. All other authors declared no competing interests for this work.

## Supporting information


Table S1

Table S2

Table S3

Table S4

Figure S1
