# Supplementary material for: Development of breakthrough bleeding model of combined‐oral contraceptives utilizing model‐based meta‐analysis
Source: CPT Pharmacometrics Syst Pharmacol. 2024 Nov 17;13(11):2016–25. doi: 10.1002/psp4.13261 (PMC11578130; doi:10.1002/psp4.13261)
Supplement: Supplementary file 1 — Table S1 [file PSP4-13-2016-s002.docx]

Table S1. Search criteria for database building.

The keywords were included during the search for hormonal contraceptives of interest, focusing on safety endpoint of breakthrough bleeding. Exclusion criteria were applied, including references with indications other than pregnancy prevention, other study types such as case-control, cohort, or open-label extension studies, studies lacking endpoints of bleeding, those without active treatment, multiphasic dosing treatment, or identified as review articles.

| "Clinical Trials" OR "Clinical trial[Publication Type]" OR "clinical trial" OR "Observational study" OR "Observational Studies as Topic" |
| --- |
| "Contraceptive Agents, Estrogen" OR "Contraceptive Agents, Oral, Hormonal" OR "Hormonal Oral Contraceptive Agents" OR "Hormonal Oral Contraceptives" OR "Low-Dose Oral Contraceptives" OR "Oral Contraceptives" OR "Oral Contraceptives, Hormonal" OR "Oral Contraceptives, Low Dose" OR "Oral Contraceptives, Low-Dose" OR "Oral Contraceptives, Phasic" OR "Phasic Oral Contraceptives" OR "Hormonal contraceptives" OR "Hormonal contraception" OR "Contraceptives, hormonal" |
| "Intermenstrual bleeding" OR "breakthrough bleeding" OR "Abnormal bleeding" OR "bleeding" OR "spotting" OR "withdrawal bleeding" OR "bleed" OR "intracyclic bleeding" OR "vaginal bleeding" OR "unscheduled bleeding" OR "unexpected bleeding" OR "menstrual bleeding" OR "irregular bleeding" |
| "Ethinyl Estradiol" OR "Estradiol, Ethinyl" OR "Ethinyloestradiol" OR "Ethynyl Estradiol" OR "Estradiol, Ethynyl" OR "Hemihydrate, Ethinyl Estradiol" OR "Progynon C" OR "Microfollin" OR "Lynoral" OR "Estinyl" |
| "Levonorgestrel"OR "l-Norgestrel" OR "l Norgestrel" OR "D-Norgestrel" OR "D Norgestrel" OR "Microval" OR "Microlut" OR "Mirena" OR "Norgeston" OR "ethinyl estradiol - levonorgestrel" OR "Microgynon" OR "Trikvilar" OR "Triregol" OR "aviane" OR "Gynatrol" |
| "Drospirenone" OR "dihydrospirorenone" OR "Slynd" OR "Yasmin" OR "Yasminelle" OR "Yaz" OR "Gianvi" OR "Nikki" OR "Loryna" OR "Ocella" OR "Zarah" OR "Syeda" OR "Nextstellis" OR "Jasmiel" OR "Zumandimine" OR "Lo-Zumandimine" |
| "Gestodene" OR "Femodene" OR "Gynera" OR "Harmonet" OR "Meliane" OR "Minesse" OR "Minulet" |
| "Norgestrel" OR "Ovral" OR "dl-Norgestrel" OR "DL-Norgestrel" OR "WY-3707" |
| "Norgestimate" OR "Cilest" OR "Ortho-Cyclen" OR "Prefest" |
| "Desogestrel" OR "Cerazette" |
| "Dienogest" OR "Dinagest" OR "Visanne" OR "Natazia" OR "Qlaira" OR "Valette" |
| "Norethindrone" OR "Norpregneninolone" OR "Norethisterone" OR "Ethinylnortestosterone" OR "Conceplan" OR "Micronor" OR "Norlutin" OR "Nor-QD" OR "Nor QD" OR "NorQD" OR "Norcolut" OR "Norcolute" OR "Monogest" |
