# Supplementary material for: Development of breakthrough bleeding model of combined‐oral contraceptives utilizing model‐based meta‐analysis
Source: CPT Pharmacometrics Syst Pharmacol. 2024 Nov 17;13(11):2016–25. doi: 10.1002/psp4.13261 (PMC11578130; doi:10.1002/psp4.13261)
Supplement: Supplementary file 2 — Table S2 [file PSP4-13-2016-s001.docx]

Table S2. Summary of database used for BTB analysis with imputed demographic covariates.

| Study ID | Arm ID | Duration [months] | EE dose [mcg] | Progestin dose [mcg] | Mean age [years] | Mean BW [kg] | Mean BMI [kg/m2] | Mean HT (SD) [cm] | Number of women | Progestin Type | Reference |
| --- | --- | --- | --- | --- | --- | --- | --- | --- | --- | --- | --- |
| 1 | 1 | 4.67 | 15 | 60 | 27.6 | 53.7 | 21.4 | 157.3 | 94 | GSD | Jaithitivit et al.^17^ |
| 2 | 1 | 5 | 30 | 3000 | 22.5 | 68.3 | 27.6 | 161.62 | 30 | DRSP | Kriplani et al.^18^ |
| 3 | 1 | 23.34 | 20 | 3000 | 24.6 | 63.4 | 22.4 | 167.98 | 461 | DRSP | Gruber et al.^19^ |
| 4 | 1 | 4.67 | 30 | 3000 | 26.8 | 58.72 | 22.8 | 164.15 | 203 | DRSP | Borges et al.^20^ |
| 5 | 1 | 10.27 | 15 | 60 | 30.13 | 62.36 | 23.35 | 164.29 | 58 | GSD | Fruzzetti et al.^21^ |
| 6 | 1 | 1.87 | 30 | 150 | 26.4 | 56.7 | 23.37 | 164.47 | 115 | DSG | Zichella et al.^22^ |
| 6 | 2 | 4.67 | 30 | 75 | 27.2 | 57.1 | 23.45 | 165.1 | 126 | GSD | Zichella et al.^22^ |
| 7 | 1 | 11.2 | 30 | 3000 | 25.2 | 60.5 | 23.25 | 166.1 | 942 | DRSP | Huber et al.^23^ |
| 7 | 2 | 11.2 | 30 | 150 | 25.3 | 59.9 | 23.43 | 166.2 | 260 | DSG | Huber et al.^23^ |
| 8 | 1 | 10.27 | 20 | 150 | 25.1 | 59.3 | 23.13 | 162.39 | 736 | DSG | Endrikat et al.^24^ |
| 8 | 2 | 10.27 | 20 | 75 | 25.5 | 59.2 | 23.06 | 161.82 | 740 | GSD | Endrikat et al.^24^ |
| 9 | 1 | 4.67 | 20 | 100 | 26 | 63 | 23.48 | 167 | 1456 | LNG | Boerrigter et al.^25^ |
| 10 | 1 | 4.67 | 20 | 100 | 25.6 | 62.7 | 23.44 | 167.1 | 805 | LNG | Bannemerschult et al.^26^ |
| 11 | 1 | 4.67 | 30 | 150 | 26.2 | 57.41 | 23.41 | 164.74 | 394 | DSG | Koetsawang et al.^27^ |
| 11 | 2 | 4.67 | 30 | 75 | 26 | 60.09 | 23.43 | 164.96 | 389 | GSD | Koetsawang et al.^27^ |
| 12 | 1 | 10.27 | 20 | 150 | 40.2 | 46.02 | 22.1 | 164.44 | 58 | DSG | Trossarelli et al.^28^ |
| 13 | 1 | 4.67 | 30 | 75 | 25.6 | 61.2 | 23.36 | 164.34 | 95906 | GSD | Brill et al.^29^ |
| 14 | 1 | 8.4 | 30 | 150 | 27.56 | 54 | 23.31 | 163.88 | 226 | LNG | Ingemanson et al.^30^ |
| 15 | 1 | 4.67 | 15 | 60 | 25 | 58.03 | 22.03 | 153.06 | 163 | GSD | Barbosa et al.^31^ |
| 16 | 1 | 11.2 | 30 | 3000 | 26.4 | 63.2 | 23.49 | 167.1 | 298 | DRSP | Parsey and Pong ^32^ |
| 17 | 1 | 4.67 | 35 | 75 | 16.4 | 57 | 23.35 | 164.23 | 5602 | GSD | Brill et al.^33^ |
| 18 | 1 | 9 | 30 | 150 | 26.6 | 56.98 | 23.31 | 163.91 | 408 | DSG | Dierendonck et al.^34^ |
| 19 | 1 | 4.67 | 30 | 75 | 26.04 | 59.8 | 23.3 | 163.85 | 229 | GSD | Loudon et al.^35^ |
| 19 | 2 | 4.67 | 30 | 150 | 26.23 | 58.7 | 23.08 | 162 | 227 | LNG | Loudon et al.^35^ |
| 20 | 1 | 9 | 30 | 150 | 26.9 | 46.73 | 23.32 | 163.98 | 600 | LNG | Ramos et al.^36^ |
| 21 | 1 | 11.2 | 20 | 100 | 25.3 | 62.9 | 23.43 | 164.94 | 380 | LNG | Endrikat et al.^37^ |
| 21 | 2 | 7.47 | 30 | 150 | 26.1 | 64.4 | 23.53 | 165.78 | 125 | LNG | Endrikat et al.^37^ |
| 22 | 1 | 1.87 | 30 | 150 | 25.46 | 52.99 | 22.08 | 154.74 | 70 | LNG | Tantbirojn and Taneepanichskul ^38^ |
| 23 | 1 | 1.87 | 30 | 3000 | 27.2 | 52.8 | 23.19 | 154.8 | 58 | LNG | Suthipongse and Taneepanichskul^39^ |
| 23 | 2 | 1.87 | 30 | 150 | 26.3 | 53.1 | 23.05 | 154.6 | 57 | LNG | Suthipongse and Taneepanichskul^39^ |
| 24 | 1 | 4.67 | 20 | 150 | 28.2 | 60.24 | 23.49 | 165.49 | 338 | DSG | Winkler et al.^40^ |
| 24 | 2 | 4.67 | 20 | 100 | 28.5 | 58.28 | 23.45 | 165.09 | 326 | LNG | Winkler et al.^40^ |
| 25 | 1 | 11.2 | 20 | 100 | 27.2 | 64.2 | 22.8 | 167.8 | 308 | LNG | Teichmann et al.^41^ |

REFERENCES

17. Jaithitivit L, Jaisamrarn U, Taneepanichskul S. Cycle control, safety and acceptability of a new oral contraceptive containing ethinylestradiol 15 micrograms and gestodene 60 micrograms. *J Med Assoc Thail Chotmaihet Thangphaet*. 2012;95(5):630-635.

18. Kriplani A, Periyasamy AJ, Agarwal N, Kulshrestha V, Kumar A, Ammini AC. Effect of oral contraceptive containing ethinyl estradiol combined with drospirenone vs. desogestrel on clinical and biochemical parameters in patients with polycystic ovary syndrome. *Contraception*. 2010;82(2):139-146. doi:10.1016/j.contraception.2010.02.009

19. Gruber DM, Huber JC, Melis GB, Stagg C, Parke S, Marr J. A comparison of the cycle control, safety, and efficacy profile of a 21-day regimen of ethinylestradiol 20mug and drospirenone 3mg with a 21-day regimen of ethinylestradiol 20mug and desogestrel 150mug. *Treat Endocrinol*. 2006;5(2):115-121. doi:10.2165/00024677-200605020-00005

20. Borges LE, Andrade RP, Aldrighi JM, et al. Effect of a combination of ethinylestradiol 30 microg and drospirenone 3 mg on tolerance, cycle control, general well-being and fluid-related symptoms in women with premenstrual disorders requesting contraception. *Contraception*. 2006;74(6):446-450. doi:10.1016/j.contraception.2005.10.016

21. Fruzzetti F, Genazzani AR, Ricci C, De Negri F, Bersi C, Carmassi F. A 12-month clinical investigation with a 24-day regimen containing 15 microg ethinylestradiol plus 60 microg gestodene with respect to hemostasis and cycle control. *Contraception*. 2001;63(6):303-307. doi:10.1016/s0010-7824(01)00213-x

22. Zichella L, Sbrignadello C, Tomassini A, et al. Comparative study on the acceptability of two modern monophasic oral contraceptive preparations: 30 microgram ethinyl estradiol combined with 150 microgram desogestrel or 75 microgram gestodene. *Adv Contracept Off J Soc Adv Contracept*. 1999;15(3):191-200. doi:10.1023/a:1006745315344

23. Huber J, Foidart JM, Wuttke W, et al. Efficacy and tolerability of a monophasic oral contraceptive containing ethinylestradiol and drospirenone. *Eur J Contracept Reprod Health Care Off J Eur Soc Contracept*. 2000;5(1):25-34. doi:10.1080/13625180008500375

24. Endrikat J, Düsterberg B, Ruebig A, Gerlinger C, Strowitzki T. Comparison of efficacy, cycle control, and tolerability of two low-dose oral contraceptives in a multicenter clinical study. *Contraception*. 1999;60(5):269-274. doi:10.1016/s0010-7824(99)00097-9

25. Boerrigter PJ, Ellman H, Dolker M. International clinical experience with a new low-dose, monophasic oral contraceptive containing levonorgestrel 100 microg and ethinyl estradiol 20 microg. *Clin Ther*. 1999;21(1):118-127. doi:10.1016/s0149-2918(00)88272-7

26. Bannemerschult R, Hanker JP, Wünsch C, Fox P, Albring M, Brill K. A multicenter, uncontrolled clinical investigation of the contraceptive efficacy, cycle control, and safety of a new low dose oral contraceptive containing 20 micrograms ethinyl estradiol and 100 micrograms levonorgestrel over six treatment cycles. *Contraception*. 1997;56(5):285-290. doi:10.1016/s0010-7824(97)00157-1

27. Koetsawang S, Charoenvisal C, Banharnsupawat L, Singhakovin S, Kaewsuk O, Punnahitanont S. Multicenter trial of two monophasic oral contraceptives containing 30 mcg ethinylestradiol and either desogestrel or gestodene in Thai women. *Contraception*. 1995;51(4):225-229. doi:10.1016/0010-7824(95)00037-b

28. Trossarelli GF, Gennarelli G, Benedetto C, et al. Climacteric symptoms and control of the cycle in women aged 35 years or older taking an oral contraceptive with 0.150 mg desogestrel and 0.020 mg ethinylestradiol. *Contraception*. 1995;51(1):13-18. doi:10.1016/0010-7824(94)00009-l

29. Brill K, Norpoth T, Schnitker J, Albring M. Clinical experience with a modern low-dose oral contraceptive in almost 100,000 users. *Contraception*. 1991;43(2):101-110. doi:10.1016/0010-7824(91)90037-g

30. Ingemanson CA, Jägerhorn M, Zizala J, Nilsson B, Zador G. Preliminary results from a swedish multicenter trial of a new low dose combined oral contraceptive. *Acta Obstet Gynecol Scand Suppl*. 1976;54:71-75. doi:10.3109/00016347609156452

31. Barbosa IC, Filho CI, Faggion D, Baracat EC. Prospective, open-label, noncomparative study to assess cycle control, safety and acceptability of a new oral contraceptive containing gestodene 60 microg and ethinylestradiol 15 microg (Minesse). *Contraception*. 2006;73(1):30-33. doi:10.1016/j.contraception.2005.06.057

32. Parsey KS, Pong A. An open-label, multicenter study to evaluate Yasmin, a low-dose combination oral contraceptive containing drospirenone, a new progestogen. *Contraception*. 2000;61(2):105-111. doi:10.1016/s0010-7824(00)00083-4

33. Brill K, Schnitker J, Albring M. Clinical experience with a modern low-dose gestodene-containing oral contraceptive in adolescents. *Adv Contracept Off J Soc Adv Contracept*. 1994;10(4):237-247.

34. Dierendonck B, Ekwempu CC, Ladipo OA, Ulasi JN, Giwa-Osagie OF. A multicenter clinical trial in Nigeria with a low-dose oral contraceptive, Marvelon. *Adv Contracept Off J Soc Adv Contracept*. 1993;9(1):25-32. doi:10.1007/BF02115897

35. Loudon NB, Kirkman RJ, Dewsbury JA. A double-blind comparison of the efficacy and acceptability of Femodene and Microgynon-30. *Eur J Obstet Gynecol Reprod Biol*. 1990;34(3):257-266. doi:10.1016/0028-2243(90)90079-g

36. Ramos R, Apelo R, Osteria T, Vilar E. A comparative analysis of three different dose combinations of oral contraceptives. *Contraception*. 1989;39(2):165-177. doi:10.1016/s0010-7824(89)80005-8

37. Endrikat J, Hite R, Bannemerschult R, Gerlinger C, Schmidt W. Multicenter, comparative study of cycle control, efficacy and tolerability of two low-dose oral contraceptives containing 20 microg ethinylestradiol/100 microg levonorgestrel and 20 microg ethinylestradiol/500 microg norethisterone. *Contraception*. 2001;64(1):3-10. doi:10.1016/s0010-7824(01)00221-9

38. Tantbirojn P, Taneepanichskul S. Clinical comparative study of oral contraceptives containing 30 microg ethinylestradiol/150 microg levonorgestrel, and 35 microg ethinylestradiol/250 microg norgestimate in Thai women. *Contraception*. 2002;66(6):401-405. doi:10.1016/s0010-7824(02)00393-1

39. Suthipongse W, Taneepanichskul S. An open-label randomized comparative study of oral contraceptives between medications containing 3 mg drospirenone/30 microg ethinylestradiol and 150 microg levonogestrel/30 microg ethinylestradiol in Thai women. *Contraception*. 2004;69(1):23-26. doi:10.1016/j.contraception.2003.08.014

40. Winkler UH, Ferguson H, Mulders J a. PA. Cycle control, quality of life and acne with two low-dose oral contraceptives containing 20 microg ethinylestradiol. *Contraception*. 2004;69(6):469-476. doi:10.1016/j.contraception.2003.12.017

41. Teichmann A, Apter D, Emerich J, et al. Continuous, daily levonorgestrel/ethinyl estradiol vs. 21-day, cyclic levonorgestrel/ethinyl estradiol: efficacy, safety and bleeding in a randomized, open-label trial. *Contraception*. 2009;80(6):504-511. doi:10.1016/j.contraception.2009.05.128
