# Supplementary material for: Development of breakthrough bleeding model of combined‐oral contraceptives utilizing model‐based meta‐analysis
Source: CPT Pharmacometrics Syst Pharmacol. 2024 Nov 17;13(11):2016–25. doi: 10.1002/psp4.13261 (PMC11578130; doi:10.1002/psp4.13261)
Supplement: Supplementary file 3 — Table S3 [file PSP4-13-2016-s005.docx]

Table S3. Mean and median simulated BTB after 1, 3 and 4 months of COC treatment across approved progestin/EE dose levels.

| BTB (%) after Month 1 | | | |
| --- | --- | --- | --- |
| Progestin type | EE dose (mcg) | Mean | Median |
| GSD | 15 | 13.7 | 11.9 |
| DRSP | 20 | 7.7 | 6.5 |
| DSG | 20 | 9.4 | 7.8 |
| GSD | 20 | 9.5 | 7.8 |
| LNG | 20 | 9.5 | 7.7 |
| DRSP | 30 | 5.2 | 4.0 |
| DSG | 30 | 6.8 | 5.0 |
| GSD | 30 | 6.9 | 5.2 |
| LNG | 30 | 6.6 | 4.9 |
| BTB (%) after Month 3 | | | |
| GSD | 15 | 9.5 | 8.2 |
| DRSP | 20 | 3.7 | 3.4 |
| DSG | 20 | 5.0 | 4.3 |
| GSD | 20 | 5.5 | 4.7 |
| LNG | 20 | 5.3 | 4.4 |
| DRSP | 30 | 1.5 | 1.3 |
| DSG | 30 | 2.6 | 1.9 |
| GSD | 30 | 3.3 | 2.4 |
| LNG | 30 | 2.7 | 1.9 |
| BTB (%) after Month 4 | | | |
| GSD | 15 | 8.6 | 7.4 |
| DRSP | 20 | 3.5 | 3.2 |
| DSG | 20 | 4.4 | 3.7 |
| GSD | 20 | 4.7 | 4.1 |
| LNG | 20 | 4.6 | 3.8 |
| DRSP | 30 | 1.3 | 1.2 |
| DSG | 30 | 2.1 | 1.5 |
| GSD | 30 | 2.7 | 1.9 |
| LNG | 30 | 2.1 | 1.6 |
