# Supplementary material for: Development of breakthrough bleeding model of combined‐oral contraceptives utilizing model‐based meta‐analysis
Source: CPT Pharmacometrics Syst Pharmacol. 2024 Nov 17;13(11):2016–25. doi: 10.1002/psp4.13261 (PMC11578130; doi:10.1002/psp4.13261)
Supplement: Supplementary file 4 — Table S4 [file PSP4-13-2016-s004.docx]

Table S4. Mean and median time to return to baseline unscheduled bleeding for approved progestin EE dose.

| Progestin type | Progestin dose (mcg) | EE dose(mcg) | Mean | Median |
| --- | --- | --- | --- | --- |
| GSD | 65 | 15 | 31.8 | 28.6 |
| DSG | 150 | 20 | 17.5 | 15.6 |
| DRSP | 3000 |  | 17.0 | 14.9 |
| GSD | 75 |  | 18.2 | 15.9 |
| LNG | 100 |  | 17.8 | 16.0 |
| DSG | 150 | 30 | 5.5 | 3.4 |
| DRSP | 3000 |  | 3.4 | 1.6 |
| GSD | 75 |  | 6.6 | 4.8 |
| LNG | 150 |  | 5.3 | 3.7 |
