# Supplementary material for: Development of breakthrough bleeding model of combined‐oral contraceptives utilizing model‐based meta‐analysis
Source: CPT Pharmacometrics Syst Pharmacol. 2024 Nov 17;13(11):2016–25. doi: 10.1002/psp4.13261 (PMC11578130; doi:10.1002/psp4.13261)
Supplement: Supplementary file 5 — Figure S1 [file PSP4-13-2016-s003.docx]

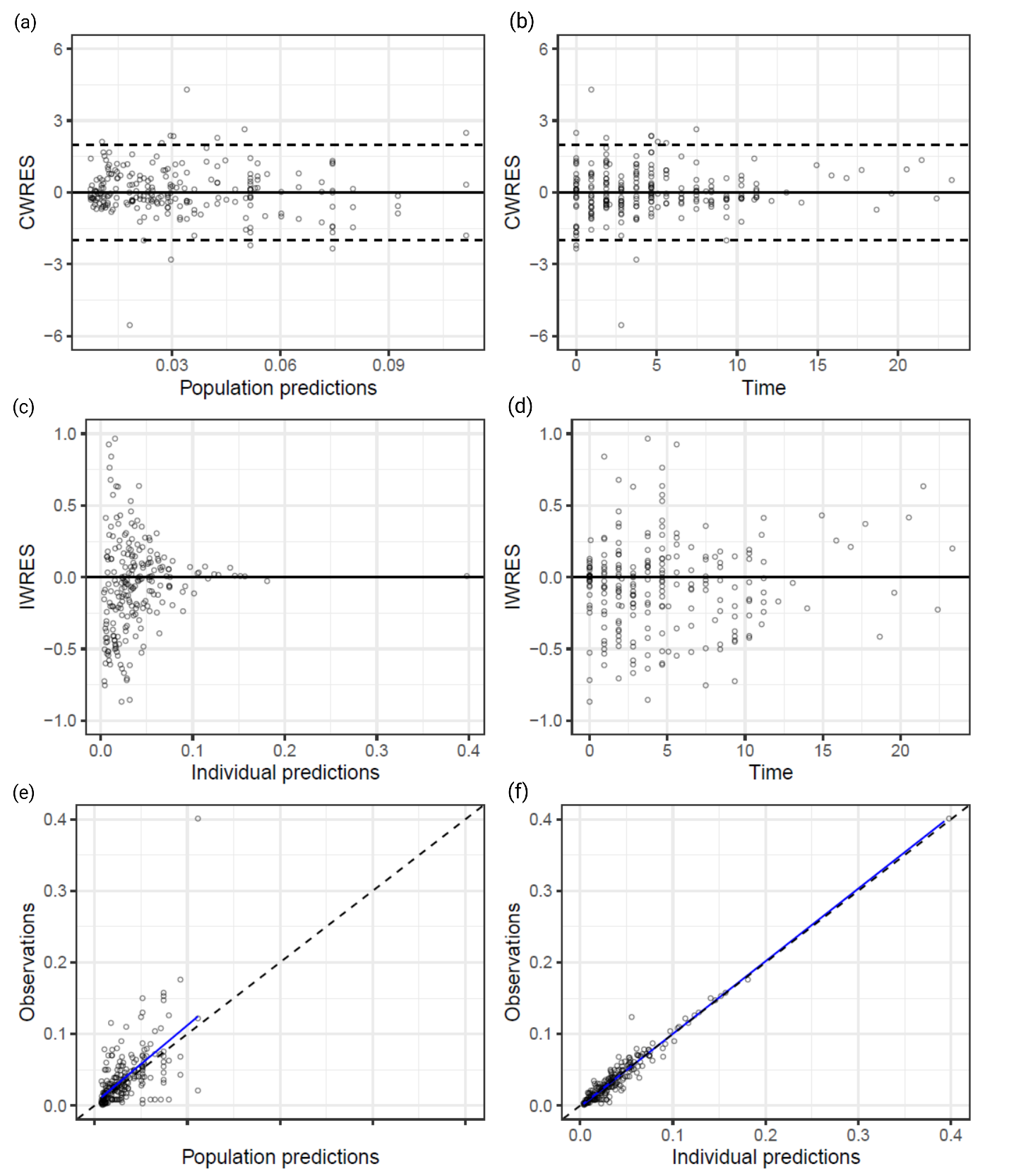


Figure S1. Basic goodness-of-fit plots for BTB. Diagnostic plots of (a) conditional weighted residuals (CWRES) versus population predictions, (b) CWRES versus time, (c) individual weighted residuals (IWRES) versus individual predictions, (d) IWRES versus time, (e) observations versus population predictions, and (f) observations versus individual predictions.
